# Large-Scale Analysis of Combining Ability and Heterosis for Development of Hybrid Maize Breeding Strategies Using Diverse Germplasm Resources

**DOI:** 10.3389/fpls.2020.00660

**Published:** 2020-06-01

**Authors:** Kanchao Yu, Hui Wang, Xiaogang Liu, Cheng Xu, Zhiwei Li, Xiaojie Xu, Jiacheng Liu, Zhenhua Wang, Yunbi Xu

**Affiliations:** ^1^College of Agriculture, Northeast Agricultural University, Harbin, China; ^2^Institute of Crop Science, Chinese Academy of Agricultural Sciences, Beijing, China; ^3^Qiqihar Branch of Heilongjiang Academy of Agricultural Sciences, Qiqihar, China; ^4^CIMMYT-China Specialty Maize Research Center, Shanghai Academy of Agricultural Sciences, Shanghai, China; ^5^CIMMYT-China Tropical Maize Research Center, Foshan University, Foshan, China; ^6^International Maize and Wheat Improvement Center (CIMMYT), Texcoco, Mexico

**Keywords:** maize, multiple-hybrid population, heterosis, heterotic groups, combining ability

## Abstract

Understanding combining ability and heterosis among diverse maize germplasm resources is important for breeding hybrid maize (*Zea mays* L.). Using 28 temperate and 23 tropical maize inbreds that represent different ecotypes and worldwide diversity of maize germplasm, we first developed a large-scale multiple-hybrid population (MHP) with 724 hybrids, which could be divided into three subsets, 325 temperate diallel hybrids and 136 tropical diallel hybrids generated in Griffing IV, and 263 temperate by tropical hybrids generated in NCD II. All the parental lines and hybrids were evaluated for 11 traits in replicated tests across two locations and three years. Several widely used inbreds showed strong general combining ability (GCA), and their derived hybrids showed strong specific combining ability (SCA). Heterosis is a quantifiable, trait-dependent and environment-specific phenotype, and the response of parental lines and their hybrids to environments resulted in various levels of heterosis. For all the tested traits except plant height and hundred grain weight (HGW), NCD II (temperate × tropical) hybrids showed higher average heterosis than the temperate and tropical diallel hybrids, with higher hybrid performance for ear length, ear diameter, and HGW. Tropical maize germplasm can be used to improve the yield potential for temperate lines. Grain number per row and grain number per ear were two most important traits that determined yield heterosis, which can be used as direct selection criteria for yield heterosis. The hybrids from heterotic groups, Reid × SPT, Reid × LRC, SPT × PA, and Lancaster × LRC, contributed highly significant positive SCA effects and strong heterosis to yield-related traits, and the heterotic patterns identified in this study were potentially useful for commercial maize breeding. Heterosis was more significantly and positively correlated with SCA than GCA, indicating that SCA can be used in heterosis prediction to develop potential hybrids in commercial maize breeding. The results of the present study not only contribute to developing breeding strategies, but also improve targeted breeding efficiency by using both temperate and tropical maize to broaden genetic basis. Large sets of parental lines with available genotypic information can be shared and used in worldwide hybrid breeding programs through an open-source breeding strategy. Potential applications of the reported results in developing hybrid maize breeding strategies were also discussed.

## Introduction

Maize (*Zea mays* L.) is one of the most important crops for staple food, livestock feed, edible oil, and biofuel (Mackay, 2009). Its cradle is in America’s tropical and subtropical areas, subsequently generating a variety of ecotypes and diverse germplasm through a process of evolution and domestication. Compared to temperate maize, those developed in tropical and subtropical zones usually have more diverse genetic variation with resistance to diseases and pests, flourishing roots, toughness stalk, lodging resistance, drought tolerance, and higher levels of heterosis in their hybrids with temperate inbreds (Vasal et al., 1992). The phenomenon of heterosis or hybrid vigor was perceived by Darwin (1876) and described as hybrid F1 offspring exhibiting phenotypic superiority than both parents (East, 1908; Shull, 1908). In plants, heterosis could be attributed to the interaction among multiple loci, depending on hybrids and traits (Schnable and Springer, 2013), as shown in the magnitude and ratio of heterosis for biomass (Li et al., 2001), flowering related traits (Krieger et al., 2010), yield (Luo et al., 2001), and resistance to abiotic and biotic stresses (Miller et al., 2015).

Utilization of heterosis is of great importance for agricultural production and one of the most successful examples in crops is from maize (Duvick, 2001). Breeding practice indicates that the performance of parents *per se* is not consistent with the hybrid performance. Excellent hybrid varieties are not necessarily derived from elite parents. Therefore, breeders should judge a parental line by its potential to produce superior hybrids, not only by its performance *per se* (Riedelsheimer et al., 2012). To identify the parental lines with great potential in making hybrids, combining ability has been estimated and used to select desirable parents and thus their hybrids. Two types of genetic parameters, GCA and SCA, have been used, which may be primarily caused by additive and non-additive gene actions, respectively (Sprague and Tatum, 1942). GCA for an inbred line is measured as the average performance for all the hybrids produced with that inbred line as the common parent, and SCA for a specific cross or hybrid is measured by the deviation of the hybrid performance from what can be predicted by the parental GCA (Sprague and Tatum, 1942). In generally, GCA evaluation is performed at early generations or breeding stages in order to save time and money in hybrid breeding, particularly in hybrid maize breeding (Sprague, 1946). The relative contribution of GCA and SCA effects to hybrid performance depends on traits and hybrids, and in some cases, for example, aflatoxin and grain yield (GY) (Meseka et al., 2018), GCA effects are more important than SCA effects. Evaluating GCA is inevitably a cumbersome and time-consuming task, becoming one of the major constraints in hybrid breeding programs.

To breed ideal hybrids with high GY (Yong et al., 2019), good quality (Machida et al., 2010) and strong resistance to biotic (Sibiya et al., 2013) and abiotic stresses (Makumbi et al., 2018), heterosis and combining ability have been analyzed for available germplasm with limited numbers of parental lines. Heterosis and combining ability are usually estimated by populations derived from special genetic designs, such as diallel (Griffing, 1956) and the NCD II (North Carolina design II) (Comstock and Robinson, 1948), which are two most powerful genetic designs for combining ability analysis and have been applied extensively. Using an NCD II with 6 × 18 parents, the combining ability analysis indicated that at least one parent with higher GCA is required for producing a hybrid cross with high SCA for nitrogen use efficiency (Cui et al., 2014). Heterotic grouping among 378 hybrids derived from diallel crosses of 28 early inbreds was evaluated for their tolerance to *Striga hermonthica*, indicating that grouping based on SCA and GCA was the most effective in classifying early maturing maize inbreds for tropical maize breeding programs (Akinwale et al., 2014). Therefore, the better understanding of the genetic basis of heterosis and combining ability we can get, the more effective maize improvement programs and hybrid performance prediction can be achieved (Dhillon and Singh, 1977). However, only limited numbers of parents and their hybrids have been used so far, with a few of exceptions that over 100 hybrids were used (Akinwale et al., 2014). The population for a hybrid crop with a large number of hybrids generated from mating designs can be simply called as multiple-hybrid population (MHP) (Wang et al., 2017). Using a specific mating design or a combination of multiple designs, large-scale MHPs can be produced for more effective analyses of combining ability and heterosis. Diverse germplasm from different ecotypes, including temperate and tropical maize, should have been used for identifying genetic variation for both basic research and commercial breeding. So far, very few analyses of combining ability and heterosis have been performed using the hybrids between different ecotypes in maize (Fan et al., 2016). Considering great genetic diversity existing in tropical maize germplasm that could contribute to further genetic improvement, more studies are required by using between-ecotype hybrids. Therefore, large-scale analysis of heterosis and combining ability using diverse germplasm resources will improve our understanding of hybrid performance significantly, thus contributing to increased genetic gain in maize hybrid breeding.

Here we report a large-scale analysis of combining ability and heterosis using an MHP consisting of 724 hybrids derived from 28 temperate and 23 tropical maize inbred lines. Our objective was to measure heterosis in both intra- and between-ecotype hybrids, estimate GCA and SCA effects and compare combining ability and heterosis across ecotypes and environments. Our results will facilitate our future maize breeding through improved combining ability analysis, defined heterotic patterns and broaden genetic basis using different maize ecotypes. The large-scale phenotypic data, combining with high-density genotypes, which can be shared and used in worldwide hybrid breeding programs through open-source breeding strategy, will provide a great opportunity for whole genome prediction of heterosis and hybrid performance.

## Materials and Methods

### Plant Materials

A maize MHP was developed by using diallel and NCD II mating designs consisting of 28 temperate and 23 tropical inbred lines, representing a broad selection of breeding germplasm from temperate and tropical regions (Wang et al., 2017). The 724 hybrids were developed, which were divided into three subsets, 325 temperate hybrids derived in Griffing IV involving 26 parental lines, 136 tropical diallel hybrids involving 17 parental lines, and 263 NCD II hybrids generated between 13 temperate and 21 tropical parental lines. Among the 28 temperate inbred lines, 15 were from China and 13 were from United States, which included six heterotic groups, Reid, SPT, LRC, Lancaster, PA, and PB (Wang et al., 2017). Among the 15 Chinese temperate lines, six are common testers, including Ye478, HZ4, Dan340, Mo17, Tie7922, and Qi319, which have been widely used in Chinese maize breeding programs. The rest nine Chinese temperate inbreds have been also playing a very important role in hybrid breeding across maize zones in China. The 23 tropical lines have been widely used as parents across worldwide breeding programs in China and CIMMYT, three of which, Jiao51, Chuan29 Female, and 18-599, are from China.

### Field Experiments and Data Collection

The temperate diallel hybrids, NCD II hybrids and their parental inbred lines were phenotyped in 3 years (2013–2015) and two locations, Shunyi, Beijing (116.6°E, 40.2°N) and Xinxiang, Henan (113.8°E, 35.1°N) in randomized block design with two replications. The tropical diallel hybrids were phenotyped in Jinghong, Yunnan (100.8°E, 22.0°N) in 2014 and Sanya, Hainan (109.2°E, 18.4°N) in 2015. In each replication, each entry was planted with two 4-m rows with 25 cm between plants and 60 cm between rows. Thinning was done at the fifth leaf stage to maintain a density of 66,600 plants/ha. Traditional agronomical practices for local maize production were adopted in each trial to manage the experimental plots.

Eleven traits of agronomic importance for hybrid performance were investigated. Two flowering related traits, days to silk (DTS) and days to anthesis (DTA) were scored. At harvesting stage, plant height (PH) and ear height (EH) were measured and seven yield-related traits, including ear length (EL), ear diameter (ED), row number (RN), grain number per row (GNPR), grain number per ear (GNPE), hundred grain weight (HGW) and grain weight per plant (GWPP), were measured after the harvested ears were air-dried. PH and EH were measured as the average height from ground to the top of the tassel and from ground to the node of the ear, respectively, each with five consecutive plants after excluding the edge ones. DTS and DTA were measured as the number of days from sowing to 50% silk and 50% anthesis, respectively. EL, ED, and RN were measured as the length from the ear bottom to tip, the diameter in the ear middle, and RN per ear, respectively. HGW was estimated with three samples of 100 kernels randomly selected from the total kernels and measured to give the average.

### Statistical Analyses

(1) SCA and GCA effects were calculated as described by Sprague and Tatum (1942) and Griffing (1956):

$$g_{i}={\bar{y}}_{i.}-{\bar{y}}_{..}$$

$$s_{i\,j}=y_{i\,j}-{\bar{y}}_{..}-g_{i}-g_{j}$$

where *g*_*i*_ and *g*_*j*_ are the GCA effects for *i*-th and *j*-th lines, respectively; *s*_*ij*_ is the SCA effect for *ij*-th hybrid; *y*_*ij*_ is the trait value of *ij*-th hybrid; ${\bar{y}}_{i.}$ is the average of the hybrids among *i*-th line crossed with a series of parents; ${\bar{y}}_{i..}$ is the overall mean.

The genetic variances of GCA and SCA effects were obtained in a joint linear mixed model analysis of MHP over all tested environments by following Riedelsheimer et al. (2012):

$$y_{i\,j\,k\,l}=\mu +L_{i}+b_{j\,\left(i\right)}+g\,c\,a_{k}+g\,c\,a_{l}+s\,c\,a_{k\,l}+L\times g\,c\,a_{i\,k}+L\times g\,c\,a_{i\,l}+L\times s\,c\,a_{i\,k\,l}+e_{i\,j\,k\,l}$$

where *y*_*ijkl*_ is the phenotypic observation for the *i*-th environment, μ is the overall mean, *L*_*i*_ is the *i*-th fixed environment effect, b*_*j*_*_(_*_*i*_*_)_ is the effect of *j*-th block within the *i*-th environment, *gca*_*k*_ and *gca*_*l*_ are the random GCA effects of the *k*-th female and the *l*-th male, *sca*_*kl*_ is the random SCA effects of the *k*-th and the *l*-th parents, *L* × *gca*_*ik*_ and *L* × *gca*_*il*_ are the random GCA by environment interaction effects, *L* × *sca*_*ikl*_ is the random SCA by location interaction effect, and *e*_*ijkl*_ is the random error.

GCA/(GCA + SCA) ratio was calculated using the equation modified from Baker (1978) by Hung and Holland (2012):

$$\frac{2\,\sigma_{G\,C\,A}^{2}}{2\,\sigma_{G\,C\,A}^{2}+\sigma_{S\,C\,A}^{2}}$$

where $\sigma_{G\,C\,A}^{2}$ is the variance of GCA effects derived from the mean square of GCA and $\sigma_{S\,C\,A}^{2}$ is the variance of SCA effects derived from the mean square of SCA. Since the total genetic variance among F1 hybrids is equal to twice the GCA component plus the SCA component, the closer this ratio is to unity, the greater the proportion of a specific hybrid’s performance can be predicted based on GCA alone (Baker, 1978).

(2) Heterosis was estimated based on two criteria, mid-parent heterosis (MPH) and high-parent heterosis (HPH), using the following formulae:

MPH(%)=100×(F1-MP)/MP

HPH(%)=100×(F1-HP)/HP

where F1 is the mean performance of F1 hybrids, MP is the parental mean, and HP is higher parent values for all tested traits.

(3) Analysis of variance (ANOVA) for phenotypic performance was performed based on combined data and linear mixed model by following Hallauer et al. (2010):

$$y_{i\,j\,k\,l}=\mu +v_{i}+y_{j}+s_{k}+{\left(v\,y\right)}_{i\,j}+{\left(v\,s\right)}_{i\,k}+{\left(y\,s\right)}_{j\,k}+{\left(v\,y\,s\right)}_{i\,j\,k}+r_{l\,\left(j\,k\right)}+e_{i\,j\,k\,l}$$

where *y*_*ijkl*_ is the *i*-th phenotypic observation for the *j*-th year, *k*-th environment, and *l*-th block, μ is the overall mean, *v*_*i*_ is the effect of *i*-th cross, *y*_*j*_ is the effect of *j*-th year, *s*_*k*_ is the effect of *k*-th environment, (*vy*)*_*ij*_* is the interaction effect between *i*-th cross and *j*-th year, (*vs*)*_*ik*_* is the interaction effect between *i*-th cross and *k*-th environment, (*ys*)*_*jk*_* is the interaction effect between *j*-th year and *k*-th environment, (*vys*)*_*ijk*_* is the interaction effect among *i*-th cross, *j*-th year, and *k*-th environment, *r*_*l*__(_*_*jk*_*_)_ is the effect of *l*-th block within *j*-th year and *k*-th environment, and *e*_*ijkl*_ is the error. The genetic effect *v*_*i*_ is considered as fixed effect while all other effects as random.

Broad sense heritability (*H*^2^) was calculated for each trait by following Knapp et al. (1985):

$$H^{2}=\frac{\sigma_{G}^{2}}{\sigma_{G}^{2}+\frac{\sigma_{G\,L}^{2}}{L}+\frac{\sigma_{E}^{2}}{L\,R}}$$

where $\sigma_{G}^{2}$ is genotypic variance of the hybrids, $\sigma_{G\,L}^{2}$ is genotype × environment interaction variance, $\sigma_{E}^{2}$ is error variance, *L* is the number of environments and *R* is the number of replications per location.

The ANOVA was performed to evaluate the effects of genotype (G), environment (E), and the interaction (G × E) using the PROC MIXED procedure of SAS^®^.^1^ Best linear unbiased predictions (BLUPs) were used to estimate phenotypic traits across multiple environments based on a linear model (Brown et al., 2011). Calculation of GCA, SCA, MPH and HPH were conducted based on the BLUP value for each trait. The correlation coefficients were assessed by the “cor.test” function in R, and the significance of the correlation coefficient was tested with *t*-test.

## Results

### Significant Effects of Genotypes and Environments on Phenotypic Traits

Phenotypic performance across environments and years with tested traits for inbreds and hybrids is shown in Table 1. Significant genotype effects were found for all measured traits (*P* < 0.01), indicating significant genetic variation among parental lines and hybrids (Supplementary Table 1). For the temperate diallel and NCD II hybrids, environment and year had significant effects on all traits except DTS and DTA, and genotype by environment effect was significant for all traits except RN. Significant genotype by year interaction (*P* < 0.01) were also found for all traits but PH and HGW. Therefore, the phenotypic performance of inbreds and hybrids was significantly affected by genotype, environment and genotype by environment interaction (Supplementary Table 1).

**TABLE 1:** Hybrids performance for tested traits in a maize multiple-hybrid population.

Trait	Temperate diallel	NCD II	Tropical diallel	MHP
PH (cm)	254.04 ± 19.11^B^	278.35 ± 13.84^A^	247.88 ± 13.70^C^	261.71 ± 20.78
EH (cm)	111.81 ± 14.65^C^	135.27 ± 13.64^A^	116.23 ± 10.34^B^	121.16 ± 17.32
DTS (d)	55.62 ± 1.81^C^	61.49 ± 2.31^B^	62.29 ± 3.17^A^	59.01 ± 3.83
DTA (d)	53.98 ± 1.87^C^	59.65 ± 2.30^B^	60.87 ± 3.02^A^	57.33 ± 3.81
EL (cm)	17.81 ± 1.40^A^	17.83 ± 1.03^A^	15.69 ± 0.60^B^	17.42 ± 1.42
ED (mm)	46.24 ± 2.41^B^	46.86 ± 2.44^A^	46.17 ± 1.87^B^	46.45 ± 2.35
RN	15.65 ± 1.21^A^	15.15 ± 1.41^B^	14.36 ± 1.08^C^	15.22 ± 1.35
GNPR	35.61 ± 3.05^A^	34.84 ± 2.90^B^	33.01 ± 1.73^C^	34.84 ± 2.95
GNPE	557.02 ± 57.57^A^	527.59 ± 58.20^B^	474.22 ± 47.40^C^	530.77 ± 63.67
HGW (g)	29.42 ± 3.27^C^	33.10 ± 3.38^A^	31.43 ± 2.55^B^	31.13 ± 3.59
GWPP (g)	131.19 ± 18.84^B^	137.43 ± 9.49^A^	135.33 ± 7.53^A^	134.23 ± 14.52

*Different superscripts letters in the same row indicate significant differences among three subsets of the multiple-hybrid population, as revealed by Tukey test at 0.01 probability. PH, plant height; EH, ear height; DTS, days to silk; DTA, days to anthesis; EL, ear length; ED, ear diameter; RN, row number; GNPR, grain number per row; GNPE, grain number per ear; HGW, hundred grain weight; GWPP, grain weight per plant. Sample sizes, temperate diallel (*N* = 325); NCD II (*N* = 263); tropical diallel (*N* = 136); MHP: multiple-hybrid population (*N* = 724).*

### Diverse Combining Ability Effects Contributed by Ecotypes and Heterotic Groups

Highly significant GCA and SCA effects were found for all tested traits (Table 2). Significant GCA × E and SCA × E interaction effects were revealed for all the traits but RN. GCA/(GCA + SCA) ratios indicate that the tested traits were predominantly controlled by additive gene effects. Broad-sense heritability estimated was high (0.77–0.93) for all traits except GWPP (0.59), suggesting that phenotypic variation observed in flowering time and yield-related traits was highly inheritable (Table 2). GCA effects were highly variable across the traits (Supplementary Table 2). In the temperate diallel, 13 temperate inbreds showed negative GCA effects for PH and EH, suggesting that these inbreds had genetic potential for reducing plant and EHs. Two inbreds, P11 (HZ1) and P22 (Qi319), showed positive GCA effects for GNPE, HGW, and GWPP. P26 (Zheng58) exhibited positive GCA effects for HGW and GWPP but negative effects for PH and EH, indicating its value in increasing GY and deceasing plant sizes. The line P2 (AS6103) showed negative effects on PH, EH and flowering related traits, with stress tolerance at early flowering stages. P19 (PH4CV) and P20 (PH6WC) had positive effects on yield traits. For 28 temperate inbred lines, American inbred lines had desirable GCA effects on HGW and GWPP, while Chinese ones had desirable GCA effects on other yield-related traits with shorter plant and EHs. For tropical parental lines, CIMMYT inbred lines had much better GCA and performance than Chinese lines. P38 (TR0415), P41 (622016-ZCN-2), P43 (CML312SR), and P48 (DTMA227B) contributed to shorter plant and EHs. P38 (TR0415), P43 (CML312SR), and P45 (CML330) contributed to early flowering. P39 (TR0423), P40 (18-599), P46 (CML504), and P50 (TR0582) contributed to higher yield-related traits. In the NCD II, temperate inbred lines had desirable GCA effects on EL, ED, HGW, DTT, and DTS, while tropical ones had more favorable effects on EH, RN, GNPR, GNPE, and GWPP.

**TABLE 2:** Analysis of variance for combining ability and heritability of tested traits in a maize multiple-hybrid population.

	PH	EH	DTS	DTA	EL	ED	RN	GNPR	GNPE	HGW	GWPP
GCA	2279.34	2231.95	91.12	89.91	8.44	40.02	10.69	44.80	19198.39	114.16	1579.68
SCA	783.69	470.45	19.03	16.62	5.68	14.02	2.75	33.34	12218.59	31.44	1632.58
GCA × E	153.84	74.73	5.09	4.14	0.64	2.05	0.36	5.59	1963.10	5.56	434.53
SCA × E	161.46	86.94	3.87	2.92	1.78	5.26	0.84^ns^	10.86	3620.95	11.55	974.28
Heritability (H)	0.88	0.92	0.91	0.93	0.79	0.85	0.88	0.77	0.80	0.87	0.59
$2\,\sigma_{G\,C\,A}^{2}/\left(2\,\sigma_{G\,C\,A}^{2}+\sigma_{S\,C\,A}^{2}\right)$	0.85	0.90	0.91	0.92	0.75	0.85	0.89	0.73	0.76	0.88	0.66

*All variance components are significant at 0.01 probability level except that labeled with “ns.” PH, plant height; EH, ear height; DTS, days to silk; DTA, days to anthesis; EL, ear length; ED, ear diameter; RN, row number; GNPR, grain number per row; GNPE, grain number per ear; HGW, hundred grain weight; GWPP, grain weight per plant. Sample sizes: multiple-hybrid population (*N* = 724).*

**FIGURE 1:**
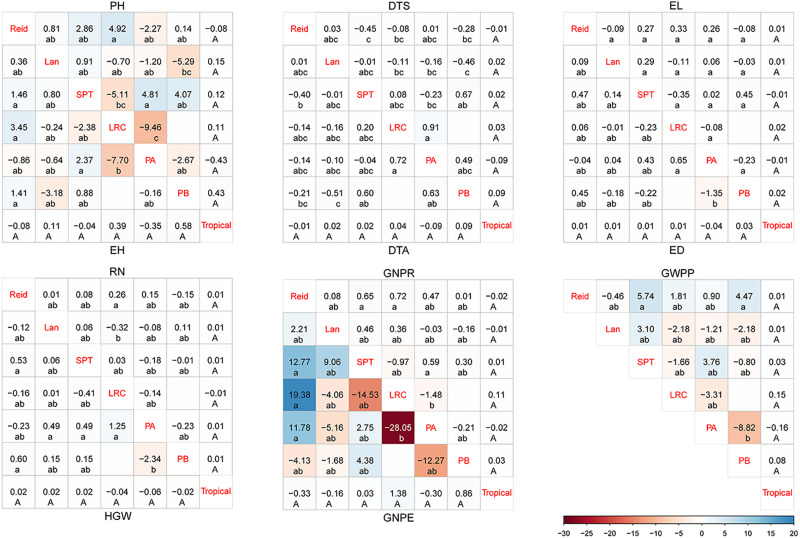
Specific combining ability (SCA) for hybrids between different heterotic groups in temperate diallel and NCD II designs. SCA is color-coded according to the color keys plotted on the bottom right. The blue and red boxes indicate positive and negative SCA effects, respectively. Different lowercase and uppercase letters indicate significant differences by Tukey test at 0.05 probability in temperate diallel and NCD II hybrids, respectively. Tropical: tropical inbred lines; Six maize heterotic groups are represented by Reid, Lan (Lancaster), SPT, LRC, PA, and PB. PH, plant height; EH, ear height; DTS, days to silk; DTA, days to anthesis; EL, ear length; ED, ear diameter; RN, row number; GNPR, grain number per row; GNPE, grain number per ear; HGW, hundred grain weight; GWPP, grain weight per plant. Sample sizes: temperate diallel (*N* = 325); NCD II (*N* = 263).

Several widely used inbreds showed higher desirable GCA effects, and SCA analysis demonstrates that the hybrids with elite materials as parents have been identified as combinations with higher desirable SCA effects (Supplementary Table 3). As shown in Figure 1 for the hybrids from different heterotic groups, the hybrids from LRC × PA showed the lowest SCA effects on PH, EH, GNPR, and GNPE but the highest SCA effects on DTS and DTA. PA × PB hybrids had the lowest SCA effects on yield-related traits, including ED, HGW, and GWPP. Reid × LRC hybrids had the highest SCA effects on PH, EH, and RN. Reid × SPT and Lan × PB hybrids had the lowest SCA effects on flowering related traits than the hybrids derived from other heterotic groups. There was no significant difference found in SCA effects for the hybrids between tropical and temperate groups. From the aforementioned analysis, Reid × SPT, Reid × PB, Reid × LRC, and SPT × PA had favorable SCA effects for yield-related traits, while Reid × SPT and Lan × PB had lower SCA effects for flowering related traits.

### Prevalent Heterosis Observed for Difference Traits

Significant MPH and HPH were observed for all the tested traits (Tables 3, 4). Two flowering related traits, DTS and DTA, exhibited negative heterosis, while others exhibited positive heterosis. For all the tested traits except HGW, a higher level of heterosis was observed in NCD II hybrids than in temperate and tropical diallel hybrids, indicating a higher level of heterosis in between-ecotype hybrids. NCD II hybrids showed a higher level of negative heterosis for flowering related traits, indicating that compared to tropical by tropical hybrids, NCD II (temperate × tropical) hybrids, with shorten growth period and improved stress tolerance, have advantages allowing them to be planted in temperate conditions. Overall, NCD II hybrids showed high levels of heterosis for EH and yield-related traits, indicating that temperate by tropical hybrids showed obviously stronger heterosis than intra-ecotype hybrids. Temperate diallel hybrids showed the lowest level of heterosis on flowering related traits, illustrating that they flowered earlier than other hybrids. The result revealed that tropical hybrids showed stronger heterosis on vegetative traits, while between-ecotype hybrids, i.e., temperate by tropical hybrids, showed a higher level of yield-related heterosis.

**TABLE 3:** Mid-parent heterosis for tested traits in a maize multiple-hybrid population.

Trait	Temperate diallel	NCD II	Tropical diallel	MHP
PH	38.47 ± 6.39^C^	50.02 ± 6.37^B^	53.18 ± 6.60^A^	45.43 ± 6.62
EH	54.14 ± 11.79^B^	65.78 ± 13.39^A^	66.47 ± 10.99^A^	60.68 ± 10.87
DTS	−6.29 ± 2.24^B^	−5.22 ± 2.39^A^	−9.81 ± 2.87	−6.56 ± 2.88
DTA	−6.85 ± 2.07^A^	−6.39 ± 2.46^A^	−10.06 ± 3.06^B^	−7.28 ± 3.07
EL	30.05 ± 9.19^B^	34.96 ± 9.57^A^	20.02 ± 6.18^C^	30.33 ± 6.14
ED	27.56 ± 4.85^B^	39.10 ± 6.21^A^	15.11 ± 4.35^C^	29.41 ± 4.34
RN	13.39 ± 5.04^B^	18.44 ± 6.85^A^	6.50 ± 5.76^C^	13.93 ± 5.77
GNPR	58.80 ± 18.46^B^	75.51 ± 19.56^A^	41.87 ± 9.38^C^	61.69 ± 9.34
GNPE	77.18 ± 24.09^B^	99.03 ± 26.39^A^	51.84 ± 15.63^C^	80.36 ± 15.65
HGW	12.14 ± 7.63^C^	28.12 ± 12.07^B^	34.76 ± 11.43^A^	22.19 ± 11.48
GWPP	141.30 ± 35.71^B^	199.54 ± 40.88^A^	136.82 ± 22.51^B^	161.61 ± 22.39

*Different superscripts letters in the same row indicate significant differences among three subsets of the multiple-hybrid population, as revealed by Tukey test at 0.01 probability. PH, plant height; EH, ear height; DTS, days to silk; DTA, days to anthesis; EL, ear length; ED, ear diameter; RN, row number; GNPR, grain number per row; GNPE, grain number per ear; HGW, hundred grain weight; GWPP, grain weight per plant. Sample sizes, temperate diallel (*N* = 325); NCD II (*N* = 263); tropical diallel (*N* = 136); MHP, multiple-hybrid population (*N* = 724).*

**TABLE 4:** High-parent heterosis for tested traits in a maize multiple-hybrid population.

Trait	Temperate diallel	NCD II	Tropical diallel	MHP
PH	30.25 ± 8.50^C^	41.84 ± 8.20^B^	45.96 ± 8.11^A^	37.41 ± 10.63
EH	37.79 ± 12.56^C^	44.69 ± 19.12^B^	55.21 ± 14.13^A^	43.57 ± 16.79
DTS	−8.91 ± 3.08^A^	−11.16 ± 4.45^B^	−13.14 ± 3.42^C^	−10.52 ± 4.03
DTA	−9.56 ± 2.83^A^	−12.62 ± 4.61^B^	−13.45 ± 3.25^B^	−11.40 ± 4.02
EL	22.20 ± 9.50^B^	25.28 ± 10.63^A^	16.73 ± 6.76^C^	22.29 ± 9.96
ED	21.93 ± 4.89^B^	28.27 ± 8.15^A^	10.90 ± 5.72^C^	22.16 ± 8.86
RN	7.07 ± 6.10^A^	9.07 ± 9.19^A^	2.26 ± 5.89^B^	6.89 ± 7.73
GNPR	48.69 ± 17.98^B^	59.10 ± 21.22^A^	35.97 ± 10.05^C^	50.08 ± 19.89
GNPE	62.63 ± 22.61^B^	73.46 ± 29.44^A^	46.81 ± 15.94^C^	63.59 ± 26.09
HGW	4.11 ± 8.74^C^	18.48 ± 12.84^B^	23.46 ± 12.64^A^	12.96 ± 13.82
GWPP	116.21 ± 33.44^B^	159.45 ± 50.57^A^	122.04 ± 21.47^B^	133.01 ± 43.83

*Different superscripts letters in the same row indicate significant differences among three subsets of the multiple-hybrid population, as revealed by Tukey test at 0.01 probability. PH, plant height; EH, ear height; DTS, days to silk; DTA, days to anthesis; EL, ear length; ED, ear diameter; RN, row number; GNPR, grain number per row; GNPE, grain number per ear; HGW, hundred grain weight; GWPP, grain weight per plant. Sample sizes: temperate diallel (*N* = 325); NCD II (*N* = 263); tropical diallel (*N* = 136); MHP, multiple-hybrid population (*N* = 724).*

### Highly Correlated Heterosis for Related Traits

**FIGURE 2:**
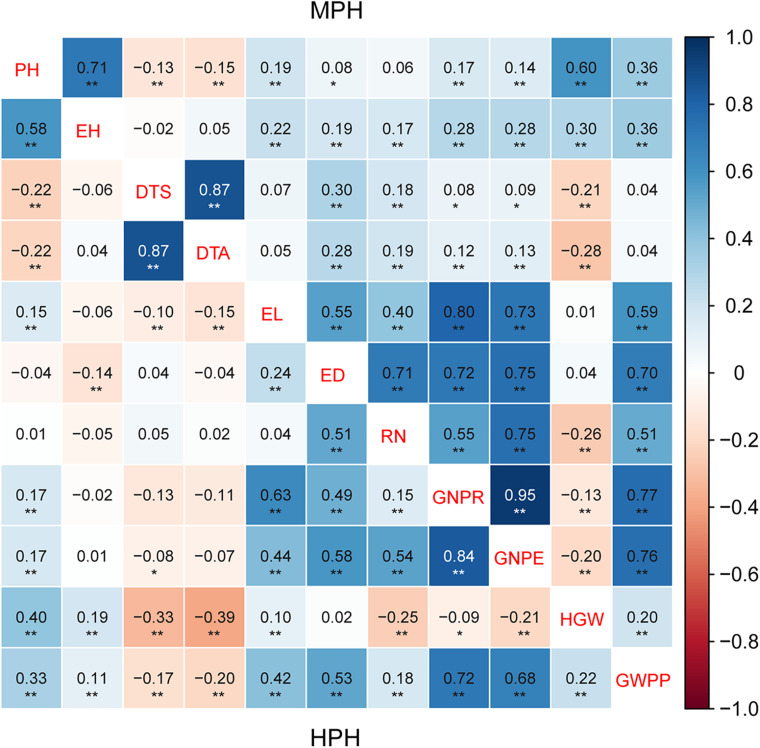
The correlation of heterosis among 11 tested traits in a maize multiple-hybrid population. Correlation coefficients for mid-parent heterosis (MPH) are listed in the upper-right, while those for high-parent heterosis (HPH) are listed in the lower-left. The correlation level is color-coded according to the color keys plotted on the right. The blue and red boxes indicate positive and negative correlation coefficients, respectively. The symbols * and ** indicate significance at 0.05 and 0.01 probability levels, respectively. PH, plant height; EH, ear height; DTS, days to silk; DTA, days to anthesis; EL, ear length; ED, ear diameter; RN, row number; GNPR, grain number per row; GNPE, grain number per ear; HGW, hundred grain weight; GWPP, grain weight per plant. Sample sizes: multiple-hybrid population (*N* = 724).

The correlation of heterosis among traits varied greatly (Figure 2). MPH showed highly significant correlation (0.87) between two flowering traits, DTS and DTA. Conversely, the correlations of the flowering traits with other traits were lower than 0.30. Yield-related traits exhibited highly significant MPH correlation with each other except HGW. A very similar trend was observed for HPH (Figure 2). Highly significant correlation was observed between MPH and HPH for all the tested traits, and the correlation for yield-related traits was higher than 0.77 (Table 5). Thus, observed heterosis largely depends on the genotype and could be correlated with related traits. Heterosis *per se* (MPH and HPH) can be treated as a phenotypic trait and used for genome selection and heterosis prediction.

**TABLE 5:** Correlation between heterosis and combining ability in a maize multiple-hybrid population.

Trait	MPH-HPH	MPH-GCA_sum_	MPH-SCA	HPH-GCA_sum_	HPH-SCA	F1-GCA_sum_	F1-SCA	F1-MPH	F1-HPH
PH	0.87**	−0.11**	0.44**	−0.17**	0.31**	0.71**	0.40**	0.24**	0.12**
EH	0.72**	−0.17**	0.47**	–0.04	0.36**	0.73**	0.35**	0.22**	0.14**
DTS	0.68**	0.02	0.50**	0.03	0.39**	0.55**	0.28**	0.02	0.19**
DTA	0.65**	0.05	0.51**	0.07	0.39**	0.56**	0.26**	–0.03	−0.21**
EL	0.84**	0.08*	0.50**	0.01	0.45**	0.67**	0.49**	0.52**	0.37**
ED	0.84**	–0.03	0.30**	−0.05**	0.33**	0.87**	0.51**	0.22**	0.18**
RN	0.77**	0.24**	0.50**	0.21**	0.48**	0.86**	0.40**	0.49**	0.43**
GNPR	0.91**	0.43**	0.38**	0.39**	0.38**	0.77**	0.59**	0.62**	0.58**
GNPE	0.89**	0.35**	0.39**	0.31**	0.39**	0.72**	0.53**	0.57**	0.51**
HGW	0.90**	0.21**	0.43**	0.22**	0.41**	0.78**	0.46**	0.60**	0.57**
GWPP	0.90**	0.04	0.39**	–0.02	0.34**	0.75**	0.67**	0.38**	0.29**

GCA_*sum*_, the sum of GCA for two parents; * and ** indicate significant at 0.05 and 0.01 probability levels, respectively. PH, plant height; EH, ear height; DTS, days to silk; DTA, days to anthesis; EL, ear length; ED, ear diameter; RN, row number; GNPR, grain number per row; GNPE, grain number per ear; HGW, hundred grain weight; GWPP, grain weight per plant. Sample sizes: multiple-hybrid population (*N* = 724).

### Heterosis Varied Significantly Across Environments

**FIGURE 3:**
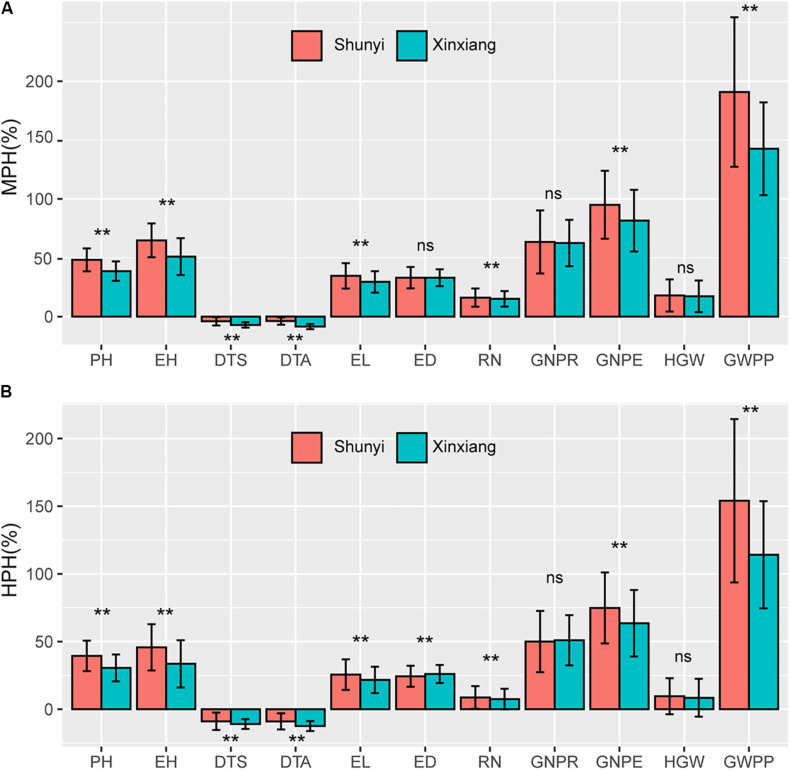
Heterosis performance in two environments, Shunyi and Xinxiang. **(A)** Mid-parent heterosis (MPH); **(B)** High-parent heterosis (HPH). In each figure red and green colors indicate Shunyi and Xinxiang, respectively. The symbols * and ** indicate significance at 0.05 and 0.01 probability levels, respectively. PH, plant height; EH, ear height; DTS, days to silk; DTA, days to anthesis; EL, ear length; ED, ear diameter; RN, row number; GNPR, grain number per row; GNPE, grain number per ear; HGW, hundred grain weight; GWPP, grain weight per plant. Sample sizes: temperate diallel (*N* = 325); NCD II (*N* = 263).

In temperate diallel hybrids and NCD II hybrids, significantly different levels of MPH were observed between two environments, Shunyi and Xinxiang, for all tested traits except ED, GNPR, and HGW (Figure 3A). Significantly different levels of HPH were also observed for all tested traits except GNPR and HGW (Figure 3B). Parents, hybrids and their combined effects contributed to the varied heterosis levels across environments. The response of maize parents and their hybrids to environmental factors may result in different levels of heterosis. We used the coefficient of variation (CV) to evaluate the stability of parents and their hybrids across the two environments. For 8 of the 11 traits, parents exhibited significantly higher CVs than hybrids (Supplementary Figure 1), indicating that for these traits the unstable heterosis across environments was driven more significantly by unstable parental lines. The rest three traits, including GNPR, HGW, and GWPP, did not exhibit significantly different CVs between hybrids and parents. These results indicate that a great variability existed in hybrids and parental lines across environments for all tested traits, and hybrids were more stable than parental lines. From the above results, environmental variables affect heterosis and hybrid performance greatly when maize hybrids and inbred lines respond differently to environmental stimuli.

### Heterotic Groups Contributed to Trait-Specific Advantages in Heterosis

**FIGURE 4:**
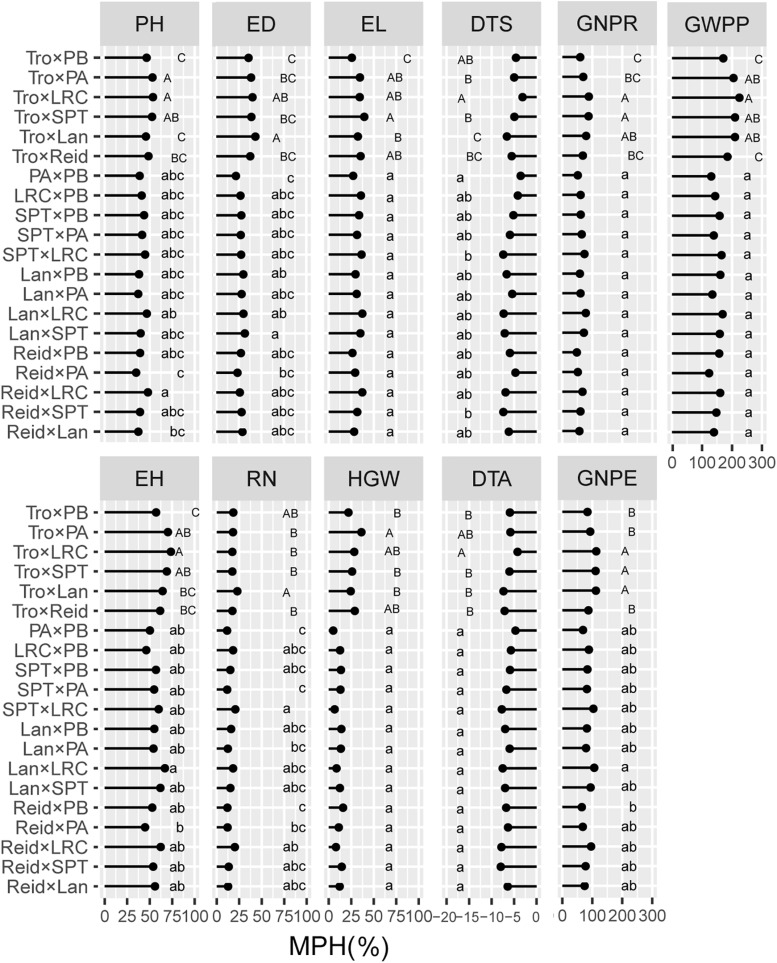
Heterosis performance in different heterotic groups for mid-parent heterosis (MPH) in temperate diallel and NCD II designs. Different lowercase and uppercase letters indicate significant differences by Tukey test at 0.05 probability in temperate diallel and NCD II hybrids, respectively. Tro, tropical inbred lines; Six maize heterotic groups are represented by Reid, Lan (Lancaster), SPT, LRC, PA, and PB. PH, plant height; EH, ear height; DTS, days to silk; DTA, days to anthesis; EL, ear length; ED, ear diameter; RN, row number; GNPR, grain number per row; GNPE, grain number per ear; HGW, hundred grain weight; GWPP, grain weight per plant. Sample sizes: temperate diallel (*N* = 325); NCD II (*N* = 263).

**FIGURE 5:**
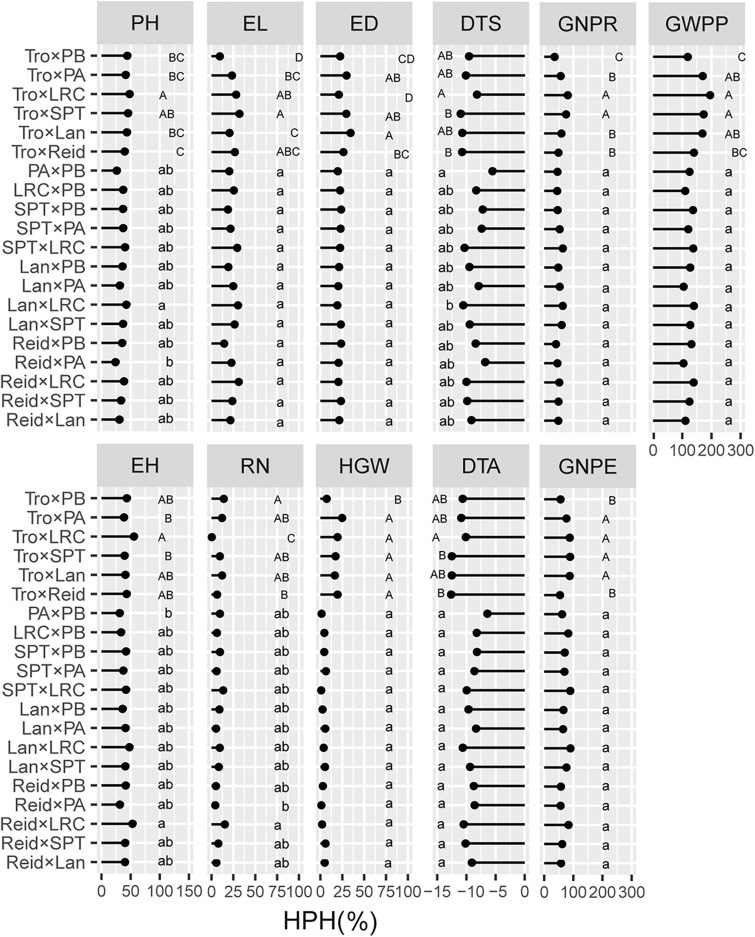
Heterosis performance in different heterotic groups for high-parent heterosis (HPH) in temperate diallel and NCD II designs. Different lowercase and uppercase letters indicate significant differences by Tukey test at 0.05 probability in temperate diallel and NCD II hybrids, respectively. Tro, tropical inbred lines; Six maize heterotic groups are represented by Reid, Lan (Lancaster), SPT, LRC, PA, and PB. PH, plant height; EH, ear height; DTS, days to silk; DTA, days to anthesis; EL, ear length; ED, ear diameter; RN, row number; GNPR, grain number per row; GNPE, grain number per ear; HGW, hundred grain weight; GWPP, grain weight per plant. Sample sizes: temperate diallel (*N* = 325); NCD II (*N* = 263).

We evaluated the average levels of heterosis across heterotic groups for the tested traits (Figures 4, 5). The Reid × LRC hybrids showed higher MPH for PH, but higher HPH for RN. The Lancaster × LRC hybrids had higher MPH for EH, and GNPE. The SPT × LRC hybrids had higher MPH for RN. Therefore, LRC group can be used to increase heterosis for yield-related traits. Both Reid × PA and Reid × PB hybrids had lower MPH and HPH for yield-related traits. SPT × LRC and Reid × SPT hybrids had lower MPH for DTS and DTA. Nonsignificant difference was found in MPH for DTA, EL, HGW, GNPR, and GWPP, or in HPH for EL, ED, HGW, DTA, GNPE, GNPR, and GWPP, among heterotic groups. Tropical × Lancaster, tropical × SPT, tropical × LRC hybrids showed higher MPH for GNPR, GNPE, and GWPP, but higher HPH for HGW, GNPE, and GWPP. Therefore, the hybrids from specific heterotic groups tended to exhibit specific trait advantages. For example, LRC group showed favorable heterosis for yield-related traits, while LRC, SPT and Lancaster groups had lower heterosis for flowering related traits.

### GNPR and GNPE as Two Important Traits for Yield Heterosis

**FIGURE 6:**
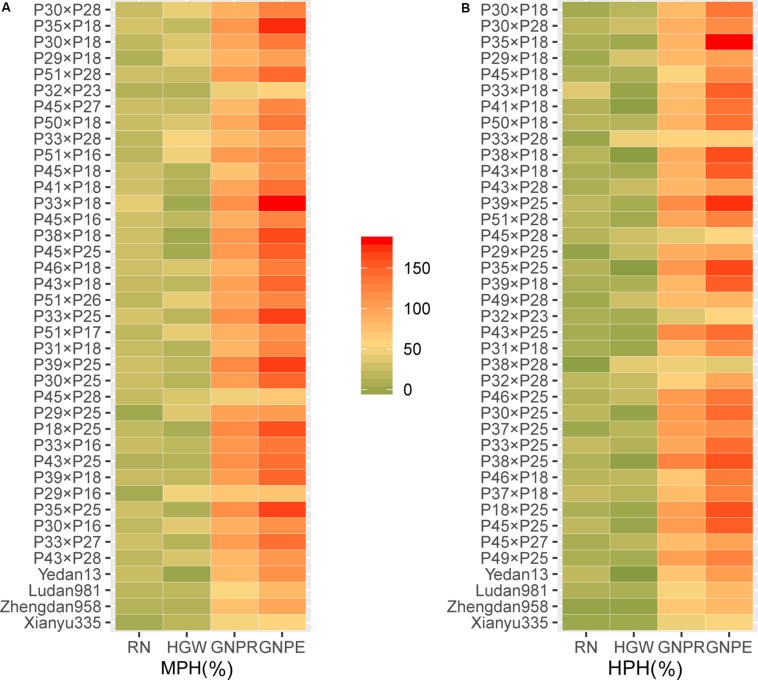
The yield-related heterosis for GWPP ranked in the top 5% hybrids and four representative commercial maize hybrids in a maize multiple-hybrid population. **(A)** mid-parent heterosis (MPH); **(B)** high-parent heterosis (HPH). The rank is in accordance with the order for heterosis of GWPP from high to low. RN, row number; GNPR, grain number per row; GNPE, grain number per ear; HGW, hundred grain weight; GWPP, grain weight per plant. Sample sizes: multiple-hybrid population (*N* = 724).

Yield was evaluated based on GWPP. The tested hybrids showed significant MPH and HPH for both yield and six yield-related traits (Tables 3, 4). GWPP in temperate diallel hybrids showed 141.30% (MPH) and 116.21% (HPH) on average over parental lines. Moreover, the hybrids showed significantly higher yield-component traits than both parents. The means and ranges of seven yield-related traits in three subset populations were shown in Supplementary Figure 4. The result indicates that apparent heterosis for yield-related traits was observed in the three types of populations. The average performance of hybrids in the temperate diallel was higher than that in the NCD II and tropical diallel hybrids for ED, RN, GNPR, GNPE, while the performance in NCD II was higher than that in other two populations for EL, ED, and HGW. Temperate × tropical hybrids exhibited better yield performance and heterosis than within-ecotype hybrids for most of yield-related traits. Maize yield is determined by GNPR, GNPE, RN, and HGW. Improvement of GNPE related traits is an effective way for breeding yield heterosis. As GNPE is the multiplication of GNPR and RN, in-depth analysis of the traits related to GNPE has important theoretical and practical impacts on hybrid breeding. However, GNPR and RN are two traits that interact with each other, and GNPE is determined by both GNPR and RN. MPH showed highly significant correlation between GNPR and GNPE (0.95), between GNPE and RN (0.75) and between RN and GNPR (0.55) (Figure 2). GNPR and GNPE were two most important traits which determine yield heterosis. Heterosis for GNPR and GNPE in the temperate diallel was 58.80 and 77.18%, respectively. Heterosis for RN and HGW (13.39 and 12.14%, respectively), was relatively low, even with negative effects (Supplementary Figures 2, 3). When compared across years and locations, heterosis for GY and yield-related traits were varied significantly. Correlation analysis of heterosis between the two environments, Shunyi and Xinxiang, indicates that heterosis for yield-related traits was significantly affected by environments. The correlation of heterosis between Shunyi and Xinxiang was more significant for GNPR and GNPE than for RN and HGW (Supplementary Figures 2, 3). As heterosis for RN and HGW was relatively low (Tables 3, 4), no steadily MPH or HPH across the environments was observed. When these observations are considered together, yield heterosis can be largely explained by two major yield-related components, GNPE and GNPR, which in most cases have to offset the negative effects of RN and HGW. By examining the top 5% hybrids ranked by MPH and HPH of GWPP and four representative commercial hybrids from China, Xianyu335 (P19 × P20), Zhengdan958 (P21 × P26), Yedan13 (P10 × P16), Ludan981 (P06 × P22) (Figure 6), significant yield heterosis (MHP and HPH) was observed in all the selected hybrids, and GNPE was a major contributor to yield heterosis. GNPR was also an important determinant for yield heterosis in most hybrids. In contrast, no consistent heterosis (MPH and HPH) across environments or hybrids was observed for RN and HGW. Thus, the results suggest that both MPH and HPH for GNPE and GNPR were highly stable or consistent across these high-yielding and commercial hybrids, whereas those for RN and HGW were case-dependent.

### Significant Correlation Observed Between Heterosis and Combining Ability

Simple linear correlation coefficients were used to reveal the relationship among MPH, HPH, GCA, SCA, and F1 hybrid performance (Table 5). MPH was highly significantly correlated with SCA, and also positively correlated with GCA for all tested traits expect PH, EH, and ED. HPH was positively correlated with the sum of parental GCAs for all the tested traits except EH, DTA, ED, and GWPP. Hybrid performance showed stronger correlation with the sum of parental GCAs than with hybrid SCA. The strong correlation between heterosis (both MPH and HPH) and SCA suggests that SCA could be used to predict hybrid performance and heterosis. In contrast, no significant correlation was found between heterosis and GCA for most tested traits. The correlation of hybrid performance with SCA was higher than that with heterosis (MPH and HPH) for PH, EH, DTA, DTS, ED, and GWPP. We found that both MPH and HPH were significantly correlated with SCA, while their correlation with the sum of parental GCAs was not consistent across the tested traits. Meanwhile, we also found that both SCA and the sum of parental GCAs were highly significantly correlated with hybrid performance, and SCA was highly significantly correlated with heterosis (MPH and HPH).

## Discussion

### Potential Application of Multiple-Hybrid Populations and Open-Source Breeding Programs

Combining ability and heterosis among maize lines and correlation between combining ability, heterosis and hybrid performance provide important insights for developing breeding strategies, defining heterotic groups, and predicting hybrid performance. Complex trait dissection and crop improvement for combining ability and heterosis need to use diverse germplasm resources and large populations. Although required phenotypic variability exists in diverse maize germplasm, most researches have been using relatively small sets of inbred lines (Fan et al., 2014; Badu-Apraku et al., 2015), largely due to the fact that the number of hybrids that can be produced increases exponentially with the increase of parental lines. The MHP used in this study, consisting of 724 hybrids, was developed with Griffing IV diallel and NCD II designs using temperate and tropical elite maize inbreds as parental lines, which is suitable for combining ability and heterosis analysis and can be used for breeding different ecotypes by taking the advantages of different maize germplasm resources. Both diallel and NCD II designs can provide detailed genetic information, including dominance-recessiveness relationships and genetic interactions. In this study, an MHP could be used to reveal useful information about combining ability, heterosis, hybrid performance and genotype × environment interaction.

On the other hand, the parental lines, which have been genotyped using 55K SNP markers and resequencing (Wang et al., 2017), can be shared with international collaborators. By inferring hybrid genotypes from their parental lines, various sets of MHPs can be developed for a specific target environment or research purpose, by sharing the 51 parental lines, from which any set of hybrids, up to 1275, can be generated. If the number of parental lines increases to 200, which is manageable for many breeding programs, up to 19,900 potential hybrids can be generated to meet the requirement of worldwide breeding programs. The genotyped parental lines can be used worldwide as proposed for open-source breeding programs (Xu et al., 2017, 2020).

### Utilization of Combining Ability and Heterosis in Hybrid Maize Breeding

A full understanding of genetic basis of heterosis and combining ability remains elusive (Birchler, 2015), which, however, does not affect the vital role heterosis and combining ability play in maize breeding. Combining heterosis in different traits such as yield-related traits and stress tolerance could improve gain yield (Fujimoto et al., 2018). As shown in the present study, heterosis varied across environments as maize hybrids and inbred lines responded differently to environmental stimuli. Although heterosis was greatly affected by environmental variables, it is a quantifiable, trait-specific phenotype. In the temperate maize with six heterotic groups, the hybrids from different heterotic groups tended to exhibit trait-specific advantages. To be commercially advantageous, a hybrid should outperform its parents with respect to agronomic traits, especially the traits related to GY. Normally, GY heterosis is an important indicator of yield potential. In the present study, we demonstrated that heterosis was contributed mainly by two outperformed yield components, GNPE and GNPR. Moreover, heterosis was compromised in few cases by the negative effects of other component traits, RN and HGW. Therefore, GNPE and GNPR can be used as direct selection criteria for yield heterosis. Based on our analysis, nine elite inbred lines, HZ1, PH4CV, PH6WC, Qi319, Zheng58, TR0423, 18-599, CML504, and TR0582, had highly significant positive GCA effects for yield-related traits, which should have contributed to the improved hybrid yield. Six inbred lines, AS6103, PH4CV, PH6WC, TR0415, CML312SR, and CML330, manifested negative GCA effects for DTS and DTA, responsible for early flowering. The hybrids between heterotic groups exhibited various levels of dominance across traits, which were inconsistent among identified heterotic loci. In the elite maize hybrid Yuyu22 (Zong3 × Yu87-1), 13 heterotic loci were identified, including three for GY, seven for EL, one for RN and two for HGW (Tang et al., 2010). Several QTL were identified for seedling weight (SW), number of kernels per plant (NK), and GY in a cross between two elite inbred lines, B73 and H99 (Frascaroli et al., 2007). Using the elite maize hybrid Zhengdan958 (Zheng58 × Chang7-2), 38 heterotic loci for ear-related traits were identified, suggesting that the combination of heterotic loci in tested hybrids was genotype-dependent (Li et al., 2017). In another report, 156 QTL, 28 pairs of epistatic loci, and 10 QTL × environment interaction regions were identified, and the inheritance of yield-related traits and their MPH in Reid (PA) × Tem-tropic I (PB) hybrids with improved heterotic pattern is trait-dependent (Yi et al., 2019). The hybrids from different heterotic groups, Reid × SPT, Reid × LRC, SPT × PA, and Lancaster × LRC, showed highly significant positive SCA effect and heterosis. Considering combining ability (GCA and SCA), performance and heterosis together will help identify the hybrid combinations with comparative advantages for maize breeding. P05 × P21 (Lancaster × SPT), P07 × P21 (Reid × SPT), P32 × P23 (Tropical × SPT), P37 × P21 (Tropical × SPT), P39 × P21 (Tropical × SPT), P45 × P27 (Tropical × LRC), and P51 × P26 (Tropical × PA) contributed a highly significant positive SCA effect to GWPP and relatively high yield, MPH and HPH (Supplementary Table 3). As a result, these hybrids could be used in breeding for high yielding and wide-adaptability. Consequently, elite inbred lines with improved combining ability and associated heterotic patterns could be explored for efficient hybrid breeding.

### Use of Diverse Germplasm From Different Ecotypes in Maize Breeding

Introduction of exotic and diverse germplasms into breeding programs is of great importance in broadening genetic variation and thus improving breeding efficiency. Tropical maize germplasm, which come from the location of maize origin, host rich genetic diversity that can be used for temperate maize breeding. With the development of molecular markers and high-efficient genotyping technologies, researches have been introgressing favorable alleles from tropical into temperate maize, as shown in GY, grain moisture content and lodging resistance (Lewis and Goodman, 2003) and resistance to maize lethal necrosis disease (Gowda et al., 2015). Introgression of temperate maize germplasm into tropical lines did not disrupt the existing heterotic groups, because the introgressed lines remained genetically inclined towards the original heterotic groups from which they were derived (Musundire et al., 2019). In the present study, we observed higher average heterosis in NCD II (temperate × tropical) hybrids than that for within-ecotype hybrids for all yield-related traits except HGW, with higher hybrid performance for EL, ED, and HGW. Introgression of favorable genes and alleles from tropical maize germplasm can be explored to broaden the genetic basis of temperate maize, improve biotic and abiotic stress tolerance, optimize heterotic patterns, and develop improved temperate-tropical hybrids (Teixeira et al., 2015). Thus, large-scale analysis of combining ability and heterosis can facilitate improving targeted breeding efficiency using different ecotypes by broadening the genetic base of commercial hybrids.

### Prediction of Heterosis and Hybrid Performance Using Combining Ability

Prediction of hybrid performance and heterosis is one of the breeders’ dreams in hybrid breeding. Although there are still some gaps in our understanding of the mechanism of heterosis, great progress has been made in predicting hybrid performance (Andorf et al., 2019). The prediction can be done through integrated information including the performance of parental lines *per se*, combining ability and heterosis along with the phenotypic data collected from field evaluation (Josue and Brewbaker, 2018). In addition, different types of omics data can be explored to improve the prediction in maize (Schrag et al., 2018). There was no association found between the performance of parental lines and heterosis in F1 hybrids, because hybrid performance depends on the nature of genetic variation (Lee et al., 2007). Hence, it can be deduced that performance of parents *per se* may not be necessarily a reliable indicator of heterosis prediction. Heterosis can be treated as a single trait in genome prediction of heterosis. We observed that SCA was significantly correlated with MPH and HPH for all the tested traits, supporting that non-additive effects are the main effect for heterosis in maize (Falconer and Mackay, 1996). In contrast, the sum of parental GCAs was either negatively or not correlated with MPH or HPH. However, the correlation of hybrid performance with the sum of parental GCAs was higher than that with heterosis. We also found that hybrid performance had stronger correlation with the sum of parental GCAs than that with SCA for all the tested traits. The GCA/(GCA + SCA) ratio indicates that all the tested traits were affected by both additive and non-additive effects, but the additive effect was more important, as reported in several maize studies for kernel rows per ear (Dehghanpour and Ehdaie, 2013), PH (Pswarayi and Vivek, 2008) and GY (Sibiya et al., 2013). Therefore, the GCA/(GCA + SCA) ratio, which is close to one, suggests that GCA is much more important than SCA in hybrid maize breeding. The inbred lines with high GCA can be used as parents to develop superior hybrids in maize breeding. Strong correlation between GCA effects and hybrid performance *per se* for most traits suggests that parental GCA may be a good indicator for predicting hybrid performance. It has been reported that the accuracy of predicted SCA effects was considerably lower than that of GCA effects (Technow, 2019). As discussed above, hybrid performance was dependent on the effects of GCA and SCA, while heterosis was dependent on SCA effects. The significant and positive correlation among SCA, MPH and HPH indicates that SCA can be used to predict MPH and HPH, which is also supported by several previous studies (Solomon et al., 2012; Ndhlela et al., 2015; Tian et al., 2017). The genetic information generated from a subset of the hybrids developed in the present study can be used to predict the whole set of hybrids with specific heterotic patterns. In genomic selection, the tested parental lines and their hybrids can be used as a training population to predict the rest potential hybrids.

## Data Availability Statement

All datasets generated for this study are included in the article/Supplementary Material.

## Author Contributions

YX conceived and designed the study. KY, HW, XL, and CX analyzed the data. KY, HW, XL, CX, ZL, XX, and JL performed the experiments. KY, ZW, and YX wrote the manuscript.

## Conflict of Interest

The authors declare that the research was conducted in the absence of any commercial or financial relationships that could be construed as a potential conflict of interest.

## Footnotes

1 http://www.sas.com
