# Supplementary material for: Large-Scale Analysis of Combining Ability and Heterosis for Development of Hybrid Maize Breeding Strategies Using Diverse Germplasm Resources
Source: Front Plant Sci. 2020 Jun 1;11:660. doi: 10.3389/fpls.2020.00660 (PMC7278714; doi:10.3389/fpls.2020.00660)
Supplement: Supplementary file 1 [file Data_Sheet_1.docx]

Supplementary Material

**Large-scale combining ability and heterosis analysis for development of hybrid maize breeding strategies using diverse germplasm resources**

**Kanchao Yu^1,2,6^, Hui Wang^2^, Xiaogang Liu^2^, Cheng Xu^2^, Zhiwei Li^2^, Xiaojie Xu^2^, Jiacheng Liu^2^,**

**Zhenhua Wang^1*^, Yunbi Xu^2,3,4,5*^**

^1^College of Agriculture, Northeast Agricultural University, Harbin 150030, Heilongjiang, China

^2^Institute of Crop Science, Chinese Academy of Agricultural Sciences, Beijing, China

^3^CIMMYT-China Specialty Maize Research Center, Shanghai Academy of Agricultural Sciences, Shanghai 201400, China

^4^CIMMYT-China Tropical Maize Research Center, Foshan University, Foshan 528231, China ^5^International Maize and Wheat Improvement Center (CIMMYT), El Batan, Texcoco, CP 56130 México

^6^Qiqihar Branch of Heilongjiang Academy of Agricultural Sciences, Qiqihar, Heilongjiang, China

*** Correspondence:**Yunbi Xu

[y.xu@cgiar.org](mailto:y.xu@cgiar.org);

Zhenhua Wang

[zhenhuawang_2006@163.com](mailto:zhenhuawang_2006@163.com)

# Supplementary Figures and Tables

## Supplementary Figures


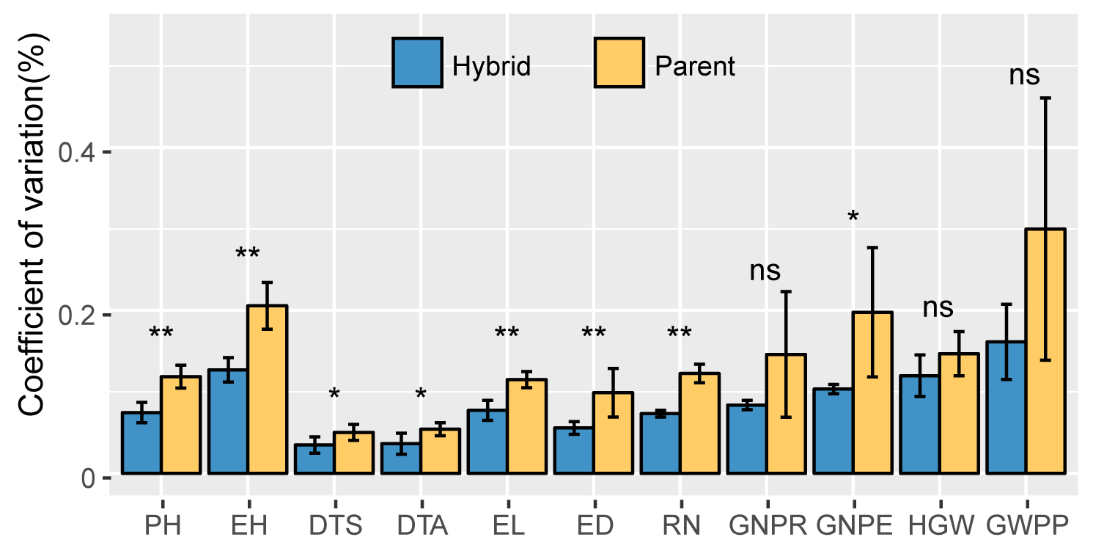


**Supplementary Figure 1** Coefficients of variation across environments for 11 tested traits compared between hybrids and parental inbreds. * and ** indicate significance at 0.05 and 0.01 probability levels, respectively. PH: plant height; EH: ear height; DTS: days to silk; DTA: days to anthesis; EL: ear length; ED: ear diameter; RN: row number; GNPR: grain number per row; GNPE: grain number per ear; HGW: hundred grain weight; GWPP: grain weight per plant. Sample sizes: temperate diallel (N=325); NCD II (N=263).


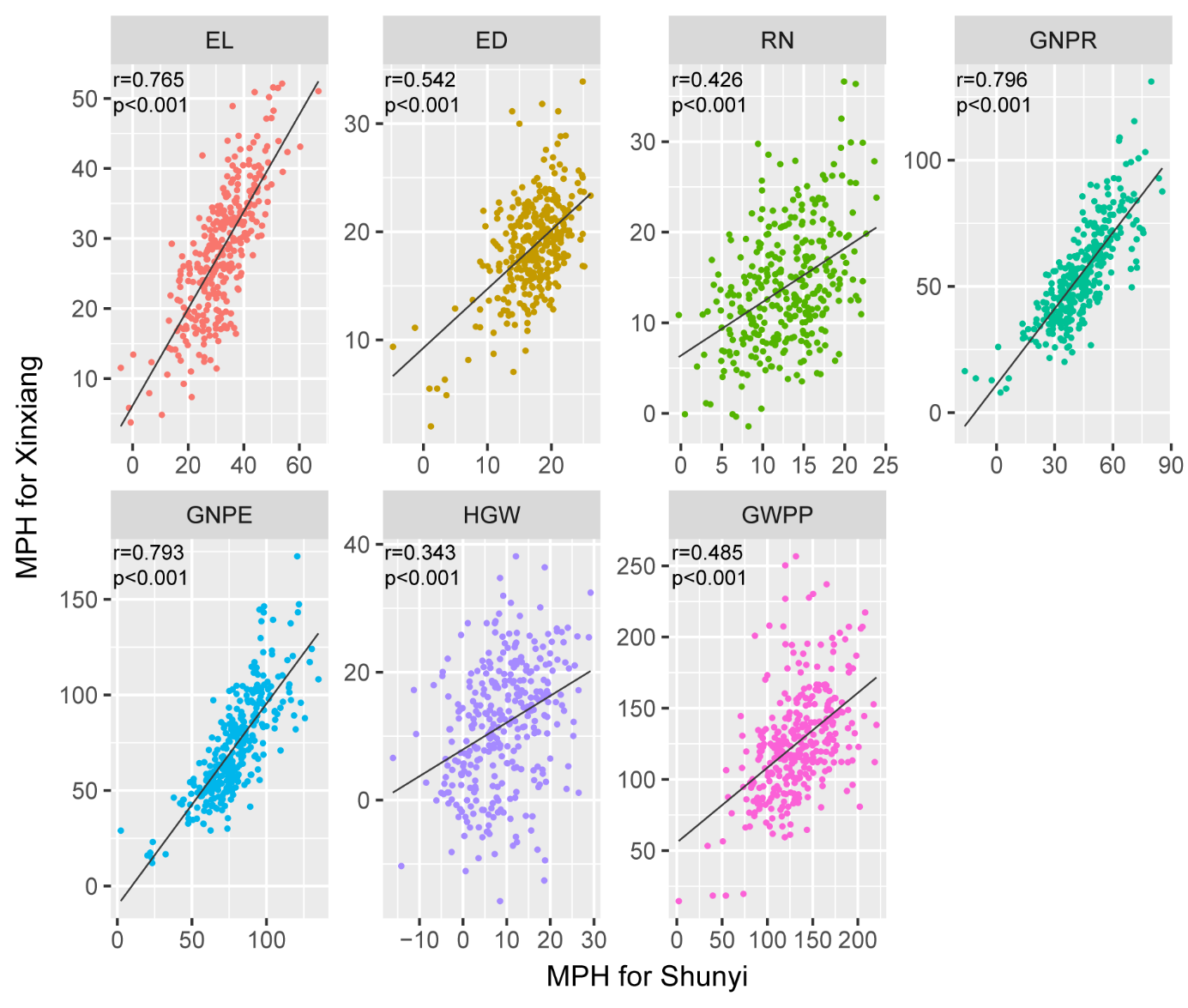


**Supplementary Figure 2** The correlation of MPH between two environments, Shunyi and Xinxiang, for the yield-related traits in temperate diallel and NCD II hybrids. EL: ear length; ED: ear diameter; RN: row number; GNPR: grain number per row; GNPE: grain number per ear; HGW: hundred grain weight; GWPP: grain weight per plant. Sample sizes: temperate diallel (N=325); NCD II (N=263).


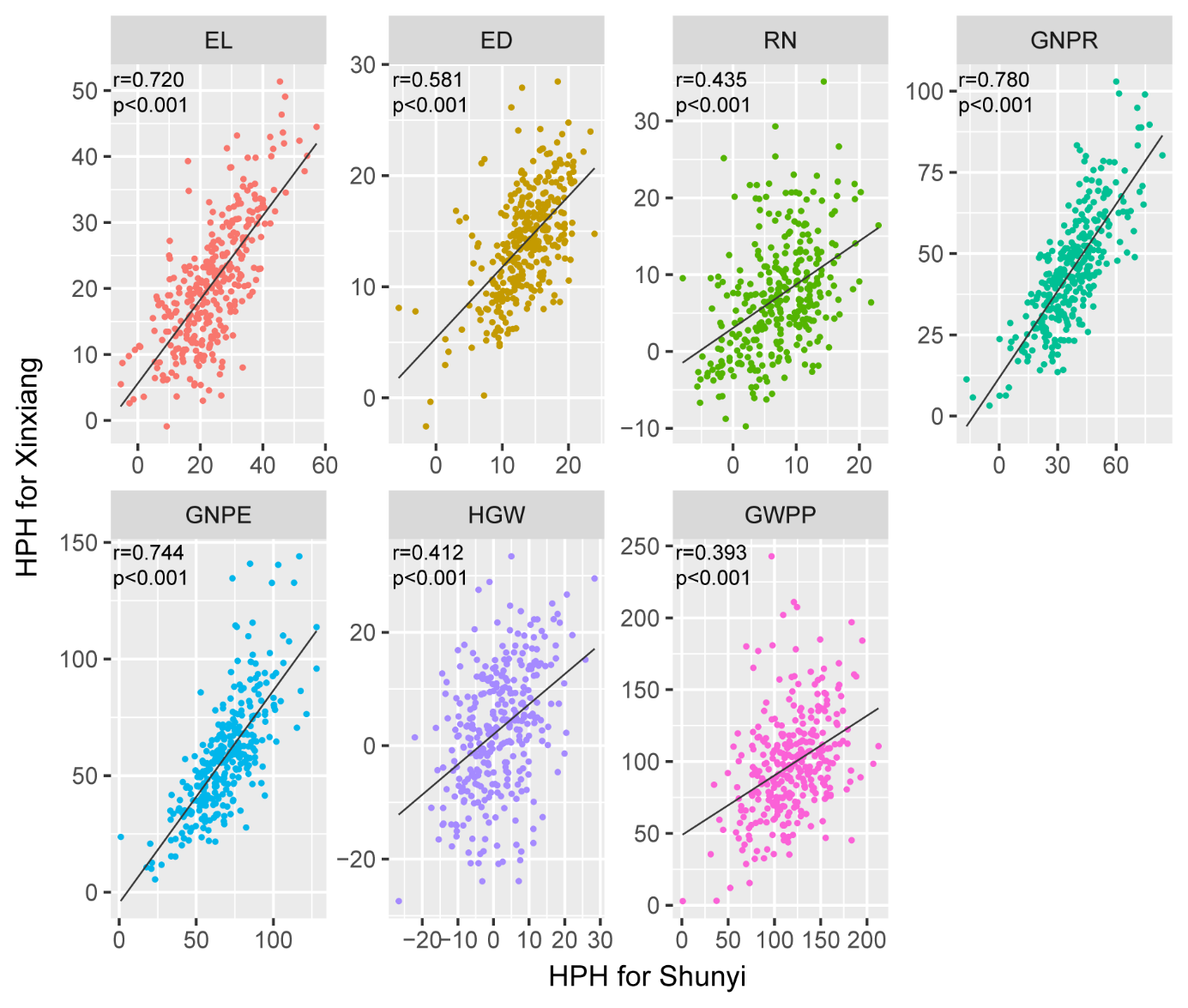


**Supplementary Figure 3** The correlation of HPH between two environments, Shunyi and Xinxiang, for the yield-related traits in temperate diallel and NCD II hybrids. EL: ear length; ED: ear diameter; RN: row number; GNPR: grain number per row; GNPE: grain number per ear; HGW: hundred grain weight; GWPP: grain weight per plant. Sample sizes: temperate diallel (N=325); NCD II (N=263).


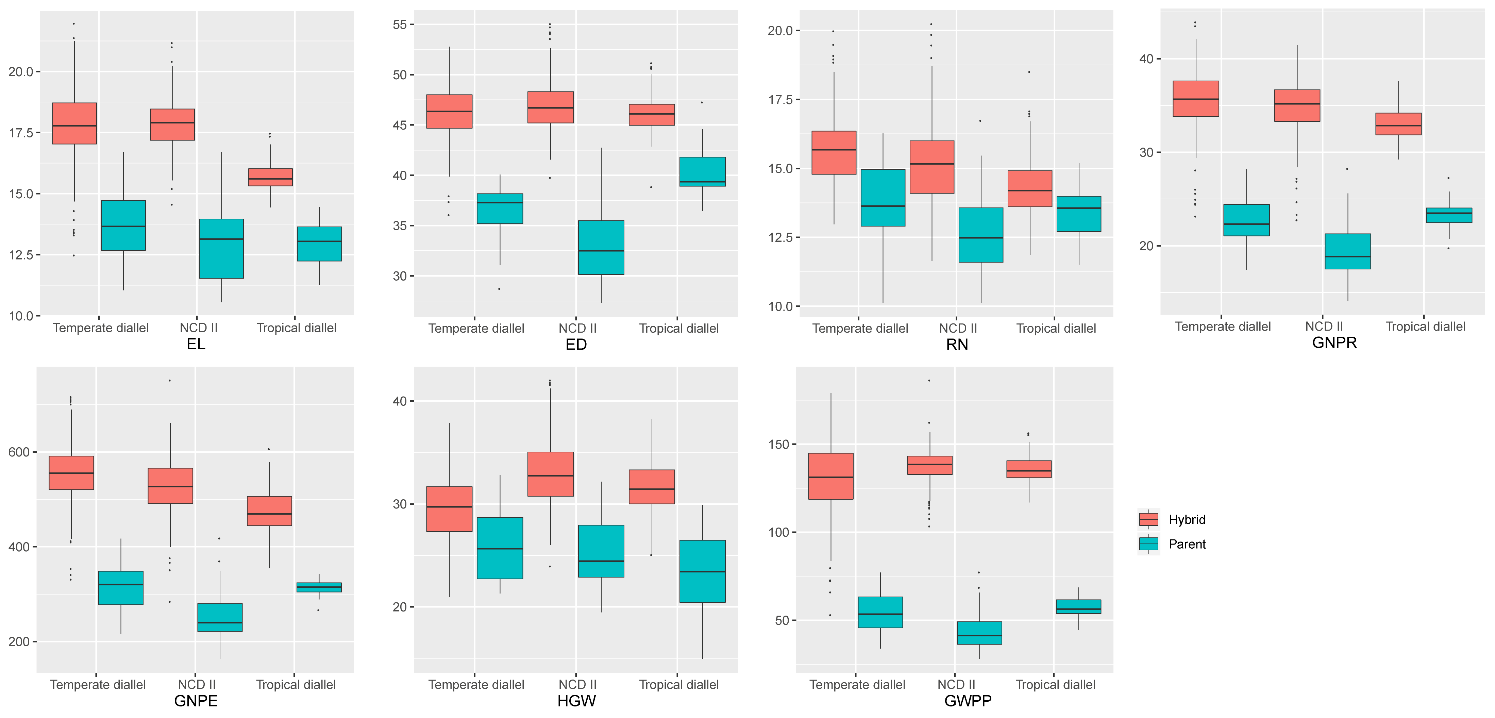


**Supplementary Figure 4** The performance of hybrids and parents for yield-related traits in a multiple-hybrid population. EL: ear length; ED: ear diameter; RN: row number; GNPR: grain number per row; GNPE: grain number per ear; HGW: hundred grain weight; GWPP: grain weight per plant. Sample sizes: temperate diallel (N=325); NCD II (N=263); tropical diallel (N=136).

# Supplementary Tables

**Supplementary Table 1** ANOVA for tested traits in a maize multiple-hybrid population with 724 hybrids.

**Supplementary Table 2** General combining ability and parental performance for 11 tested traits in a maize multiple-hybrid population with 724 hybrids.

**Supplementary Table 3** Special combining ability, hybrid performance and heterosis for 11 tested traits in a maize multiple-hybrid population with 724 hybrids.
