# Supplementary material for: Large-Scale Analysis of Combining Ability and Heterosis for Development of Hybrid Maize Breeding Strategies Using Diverse Germplasm Resources
Source: Front Plant Sci. 2020 Jun 1;11:660. doi: 10.3389/fpls.2020.00660 (PMC7278714; doi:10.3389/fpls.2020.00660)
Supplement: Supplementary file 2 [file Table_1.docx]

**Supplementary Table 1** ANOVA for tested traits in a maize multiple-hybrid population with 724 hybrids.

| Source of variation | df | PH | EH | DTS | DTA | EL | ED | RN | GNPR | GNPE | HGW | GWPP |
| --- | --- | --- | --- | --- | --- | --- | --- | --- | --- | --- | --- | --- |
| Temperate diallel  & NCD II |  |  |  |  |  |  |  |  |  |  |  |  |
| Genotype (G) | 586 | 4048.2 | 2835.9 | 107.6 | 105.7 | 18.9 | 70.0 | 16.2 | 102.7 | 38812.8 | 319.3 | 4605.8 |
| Environment (E) | 1 | 811952.9 | 758004.8 | 1833.8^ns^ | 46.0^ns^ | 1033.6^ns^ | 92.8 | 580.5 | 1271.4 | 2549395.5 | 15230.5 | 1457045.6 |
| Year (Y) | 2 | 896369.3 | 92165.5 | 21539.6^ns^ | 19312.7^ns^ | 111.6 | 685.3 | 235.2 | 668.5 | 887047.6 | 6517.9 | 888553.0 |
| G×E | 581 | 658.2 | 262.0 | 20.5 | 19.1 | 4.2 | 13.9* | 1.7^ns^ | 23.9 | 7527.2 | 251.2 | 1875.7 |
| G×Y | 796 | 316.4^ns^ | 158.4 | 18.0* | 18.4 | 3.8 | 19.0 | 2.2 | 21.5 | 7876.1 | 99.0^ns^ | 2008.4 |
| Error | 3132 | 421.9 | 136.8 | 16.2 | 15.6 | 3.3 | 12.5 | 1.6 | 15.4 | 5205.9 | 154.2 | 1385.7 |
| Tropical diallel |  |  |  |  |  |  |  |  |  |  |  |  |
| Genotype | 135 | 1007.5 | 559.3 | 437.8 | 417.3 | 17.3 | 146.3 | 14.7 | 98.4 | 28285.5 | 107.0 | 2199.8 |
| Error | 252 | 106.9 | 57.6 | 44.9 | 41.1 | 1.1 | 3.0 | 0.5 | 7.2 | 2016.0 | 5.8 | 346.0 |

*, unmarked and “ns” indicate significances at 0.05 and 0.01 probability levels and no significance, respectively. PH: plant height; EH: ear height; DTS: days to silk; DTA: days to anthesis; EL: ear length; ED: ear diameter; RN: row number; GNPR: grain number per row; GNPE: grain number per ear; HGW: hundred grain weight; GWPP: grain weight per plant. Sample sizes: temperate diallel (N=325); NCD II (N=263); tropical diallel (N=136).
